# TsImpute: an accurate two-step imputation method for single-cell RNA-seq data

**DOI:** 10.1093/bioinformatics/btad731

**Published:** 2023-12-01

**Authors:** Weihua Zheng, Wenwen Min, Shunfang Wang

**Affiliations:** Department of Computer Science and Engineering, School of Information Science and Engineering, Yunnan University, Kunming 650504, China; Department of Computer Science and Engineering, School of Information Science and Engineering, Yunnan University, Kunming 650504, China; Yunnan Key Laboratory of Intelligent Systems and Computing, Yunnan University, Kunming 650504, China; Department of Computer Science and Engineering, School of Information Science and Engineering, Yunnan University, Kunming 650504, China; Yunnan Key Laboratory of Intelligent Systems and Computing, Yunnan University, Kunming 650504, China

## Abstract

**Motivation:**

Single-cell RNA sequencing (scRNA-seq) technology has enabled discovering gene expression patterns at single cell resolution. However, due to technical limitations, there are usually excessive zeros, called “dropouts,” in scRNA-seq data, which may mislead the downstream analysis. Therefore, it is crucial to impute these dropouts to recover the biological information.

**Results:**

We propose a two-step imputation method called tsImpute to impute scRNA-seq data. At the first step, tsImpute adopts zero-inflated negative binomial distribution to discriminate dropouts from true zeros and performs initial imputation by calculating the expected expression level. At the second step, it conducts clustering with this modified expression matrix, based on which the final distance weighted imputation is performed. Numerical results based on both simulated and real data show that tsImpute achieves favorable performance in terms of gene expression recovery, cell clustering, and differential expression analysis.

**Availability and implementation:**

The R package of tsImpute is available at https://github.com/ZhengWeihuaYNU/tsImpute.

## 1 Introduction

Single-cell RNA sequencing (scRNA-seq) allows us to analyze gene expression patterns at a single-cell level and provides valuable insights into cell heterogeneity. Although scRNA-seq has been successfully applied to a variety of research tasks such as cell population identification (Petegrosso *et al.* 2019), differential expression (DE) analysis (Chung *et al.* 2021), pseudo-time analysis (Liu *et al.* 2017), and gene regulatory network inference (Pratapa *et al.* 2020), the technical limitations of scRNA-seq lead to numerous false zeros in the expression matrix, which are also called “dropouts.” The existence of dropouts distorts the information in the data and hinders downstream analysis (Wang *et al.* 2022), hence it has been recognized as a grand challenge in single-cell data analysis (Kiselev *et al.* 2019, Lähnemann *et al.* 2020).

To address the issue of dropouts, lots of studies have focused on scRNA-seq data imputation in recent years (Patruno *et al.* 2020). One main category of imputation methods is based on data smoothing or clustering. For example, DrImpute (Gong *et al.* 2018) identifies similar cells based on clustering and impute the likely dropouts by averaging expression levels of cells from the same cluster, and it adopts different distance metrics and different numbers of clusters to generate robust results. MAGIC (Dijk *et al.* 2018) constructs a Markov affinity matrix, based on which the “soft clustering” is performed and the original expression values are replaced by the weighted mean in the same cluster. ScHinter (Ye *et al.* 2019) uses an ensemble distance metric to calculate cell–cell similarities and iteratively computes the imputed values by borrowing information from similar cells. Another type of imputation methods is based on some specific statistical distributions. ScImpute (Li and Li 2018) uses a Gamma–Gaussian mixture model to estimate the dropout probability and imputes likely dropout values through non-negative least squares regression. SAVER (Huang *et al.* 2018) adopts a Poisson–Gamma mixture to model the unique molecular identifier counts and uses Poisson Lasso regression for imputation. BayNorm (Tang *et al.* 2019) assumes that the true gene expression follows negative binomial distribution and imputes the observed expression matrix through an empirical Bayes approach. ScDoc (Ran *et al.* 2020) uses Poisson-negative binomial mixture model to identify likely dropouts and impute them by borrowing information from similar cells calculated by weighted cosine similarity. Matrix decomposition approaches are also used in scRNA-seq data imputation. scRMD (Chen *et al.* 2020) models the dropout imputation problem as robust matrix decomposition and imputes the data through minimizing the reconstruction loss regularized by a nuclear norm penalty. ALRA (Linderman *et al.* 2022) uses the singular-value decomposition to compute a low-rank approximation of the observed expression matrix and sets all entries that are smaller than a threshold to zeros. WEDGE (Hu *et al.* 2021) imputes gene expression matrix by using a biased low-rank matrix decomposition methods. ScMOO (Jin *et al.* 2022) decomposes the expression matrix into three types of structures and performs imputation using multi-objective optimization.

Although a series of imputation methods have been developed from different perspectives, some limitations still remain. As is mentioned above, it is straightforward to leverage information from similar cells and generate imputed results, which involves identifying similar cells by clustering and aggregating gene expression levels of these cells. However, as dropouts are prevalent in raw scRNA-seq data, clustering results based on these noisy raw data are inaccurate and so are the imputed values (Chen *et al.* 2020, Xu *et al.* 2020). Besides, lots of imputation methods tend to alter all zeros in the expression matrix without distinguishing dropouts from true zeros, which may introduce new biases (Li and Li 2018) and take the risk of over-imputation (Patruno *et al.* 2020, Jin *et al.* 2022). Although some methods use specific statistical distribution distinguish dropouts from true zeros before imputation, most of them calculate the dropout probabilities merely for identifying whether some entry is a dropout value and rarely make full use of the statistical information.

To address issues mentioned above, we propose a novel two-step method, named tsImpute, to effectively identify and impute dropouts in scRNA-seq data. As its name implies, tsImpute performs imputation in a two-step manner: (i) first, tsImpute distinguishes likely dropouts from true zeros by estimating the parameters of zero-inflated negative binomial (ZINB) distribution, and then performs initial imputation on the likely dropouts by combining dropout probability, library size, and expected expression level; (ii) second, final imputation is performed on the preliminarily imputed matrix using inverse distance weighted (IDW) clustering, which avoids the noise in raw expression matrix. By conducting experiments on both simulated and real data, tsImpute is compared with several state-of-the-art imputation methods including ALRA, scRMD, scMOO, scImpute, SAVER, DrImpute, and MAGIC, which belong to the three categories mentioned above. Extensive experiments including data masking, cell clustering, DE analysis, and GO terms analysis show that tsImpute is able to recover biological information of scRNA-seq data and improve downstream analysis.

## 2 Materials and methods

**Figure 1.**
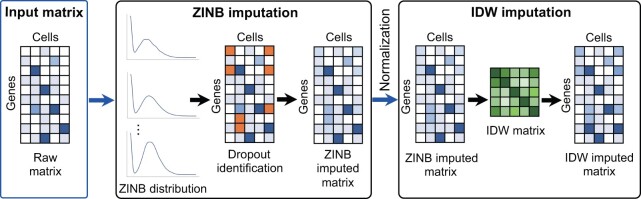
Flowchart of tsImpute. TsImpute first identifies dropouts by estimating the dropout probability of ZINB distribution, then performs initial ZINB imputation on each dropout by combining dropout probability, expression expectation of the gene, and scale factor of the cell. After initial ZINB imputation, tsImpute divides the cells into different subpopulations using hierarchical clustering and perform IDW imputation for each cluster, which borrows information from cells in the same cluster and weight the information according to the distance between the cells.

The flowchart of tsImpute is presented in Fig. 1. First, tsImpute identifies likely dropouts in the raw expression matrix with ZINB distribution and performs initial imputation using both the estimated parameters of each gene and library size of each cell. Second, tsImpute calculates the Euclidean distance matrix based on the imputed expression matrix and adopts inverse distance weighed imputation to conduct the final imputation. Pseudo-codes of tsImpute are shown in Section 7 of Supplementary Material.

### 2.1 Step 1: ZINB imputation

Consider a *m* by *n* count matrix **X**, where *m* and *n* are numbers of genes and cells, respectively. To perform initial ZINB imputation, we first estimate the dropout probability of each gene with ZINB distribution which has been successfully applied in the depiction of scRNA-seq data (Miao *et al.* 2018, Risso *et al.* 2018, Tian *et al.* 2019, 2021). ZINB distribution takes the form


(1) $$f_{\text{ZINB}}(x|\pi ,r,p)=\pi I_{0}(x)+(1-\pi )\left(\begin{array}{c} x+r-1\\ x \end{array}\right)p^{r}{\left(1-p\right)}^{x},$$

where π denotes the dropout rate; $I_{0}(x)$ is the indicator function which equals 1 when x=0 and 0 otherwise; *r* and *p* are parameters of negative binomial distribution.

Considering the heterogeneity of cells, we first divide the cells into several groups and estimate the parameters separately. However, as we have mentioned above, cell clustering based on original expression matrix tends to be influenced by dropouts. Since genes with low expression levels are more likely to be influenced by dropouts, here we use only the information of highly expressed genes for clustering: for each cell, set the expression values of the 200 highest expressed genes as 1 and other genes as 0. By this means, the cells are converted into sparse binary vectors, and now we are able to divide cells into different subpopulations according to their co-expressed genes. Specifically, we achieve this goal by conducting hierarchical clustering based on Jaccard distance: denote highly expressed gene set in cell *a* and cell *b* by $G_{a}$ and $G_{b}$, respectively, then the Jaccard distance between cell *a* and cell *b* can be expressed as


(2) $$d_{J}(a,b)=\frac{\left|G_{a}\cup G_{b}|-|G_{a}\cap G_{b}\right|}{\left|G_{a}\cup G_{b}\right|}.$$

After dividing the cells into different groups, tsImpute estimates the ZINB parameters of genes in each cell subpopulation. As ZINB is a mixture distribution, it is difficult to estimate the hidden variable, i.e. π, hence we use expectation maximization algorithm (Dempster *et al.* 1977) to estimate the parameters in an iterative manner. Given the estimates of π,r, and *p*, it is now possible to estimate the posterior dropout probability of each gene through a Bayesian approach:


(3) P(dropout|Xij=0)=P(Xij=0|dropout)⋅P(dropout)P(Xij=0)=1⋅πiP(Xij=0),

in which $P(X_{ij}=0)$ can be estimated by the proportion of zero values in gene *i*.

To make full use of the information derived from ZINB estimates, we further take into consideration the expected expression level of non-zero values in gene *i*, which can be expressed as $\frac{r_{i}(1-p_{i})}{p_{i}}$. Besides, as the expression levels of different cells vary, tsImpute also uses a scale factor to adjust the ZINB imputed values, defined as


(4) $$s_{j}=\frac{n\cdot \sum_{i=1}^{m}X_{ij}}{\sum_{i=1}^{m}\sum_{j=1}^{n}X_{ij}}.$$

Overall, given a dropout threshold *t*, the ZINB imputation value of gene *i* in cell *j* can be expressed as


(5) $$X_{ij}^{\text{init}}=\{\begin{array}{cc} \frac{\pi_{i}}{P(X_{ij}=0)}\cdot \frac{r_{i}(1-p_{i})}{p_{i}}\cdot s_{j}, & \text{if}\;\frac{\pi_{i}}{P(X_{ij}=0)}\geq t,\\ X_{ij}, & \text{otherwise}. \end{array}$$

### 2.2 Step 2: Inverse distance weighted imputation

After initial ZINB imputation, every likely dropout in the original expression matrix is now filled with a preliminarily imputed value according to both the estimated distribution of genes and the library size of cells. It is now possible to calculate reliable similarity metrics based on this modified expression matrix. In the final imputation step, we first use UMAP (McInnes *et al.* 2018) to generate low dimension representation of the cells. After dimension reduction, the initially imputed cells are divided into several groups through hierarchical clustering. It is straightforward that the expression levels of a cell should be more similar to its neighboring cells and less similar to distant cells, hence it is necessary to use a weighted scheme instead of simply averaging expression values. In tsImpute, we use a simple but effective method, i.e. IDW method (Lu and Wong 2008) to further impute the likely dropouts identified in ZINB imputation step: consider the expression sub-matrix $X^{k}$ which contains $n_{k}$ cells in some cluster *k*, we first calculate the Euclidean distance matrix $D^{k}={\left(d_{ij}\right)}_{n_{k}\times n_{k}}$, then the inverse distance weight matrix can be denoted by


(6) $$W^{k}=\left[\begin{array}{cccc} w_{11} & w_{12} & \cdots & w_{1n_{k}}\\ w_{21} & w_{22} & \cdots & w_{2n_{k}}\\ \vdots & \vdots & \ddots & \vdots \\ w_{n_{k}1} & w_{n_{k}2} & \cdots & w_{n_{k}n_{k}} \end{array}\right],$$

where $w_{ij}=\frac{1}{{\left(d_{ij}\right)}^{\alpha }}÷\sum_{j}\frac{1}{{\left(d_{ij}\right)}^{\alpha }}$, $i,j=1,\ldots ,n_{k},i\neq j,w_{ii}=\max\{w_{i\cdot }\}$ and α is a weight parameter controlling the decreasing speed of weight as the distance increases, of which the default value is set as 2. For each entry in $X^{k}$, the final imputed value can be expressed by


(7) $$X_{ij}^{\text{final}}=\{\begin{array}{cc} \sum_{j=1}^{n_{k}}w_{ij}X_{ij}^{\text{init}}, & \text{if}\;X_{ij}\;\text{is}\;a\;\text{dropout},\\ X_{ij}, & \text{otherwise}. \end{array}$$

### 2.3 Parameter selection

There are two main parameters in tsImpute, namely the number of top genes in Jaccard clustering step and dropout quantile in initial ZINB imputation which determines the proportion of genes to be imputed. The default number of top genes is set as 200 to retain only the highest expressed genes and avoid possible dropouts of the genes with a moderate expression level, but users of tsImpute can also alter this parameter to control the sparsity of binary cell vectors. As for the dropout quantile, it has been pointed out that in bulk-seq RNA data, the proportion of zeros is about 15%–40% (Jiang *et al.* 2022), while in scRNA-seq data, the zero proportion can be as high as 99% (Andrews *et al.* 2021), meaning that a large part of zeros observed in scRNA-seq data are dropouts. Hence, in tsImpute, the dropout quantile is set as 0.2 in order to identify most of the possible dropouts. Although tsImpute with default parameters works well, users of tsImpute can adjust parameters according to their own requirements, and a practical method is to tune parameters by optimizing metrics such as silhouette coefficient (Rousseeuw 1987).

## 3 Results

In this article, we compare tsImpute with seven widely used methods to evaluate its imputation performance, i.e. SAVER (Huang *et al.* 2018), DrImpute (Gong *et al.* 2018), scImpute (Li and Li 2018), MAGIC (Dijk *et al.* 2018), scRMD (Chen *et al.* 2020), ALRA (Linderman *et al.* 2022), and scMOO (Jin *et al.* 2022). To comprehensively evaluate the imputation accuracy of these methods, we conduct four different experiments to assess the performance: (i) imputation of simulated data, (ii) real data masking experiment, (iii) cell clustering, and (iv) DE analysis. In addition, as tsImpute consists of two steps of imputation, i.e. initial ZINB imputation and IDW imputation, we also conduct an ablation study to validate the significance of each step.

### 3.1 Simulation analysis

In this section, we first generate several simulated data with the widely used R package Splatter (Zappia *et al.* 2017) and compare the imputation performance of different methods. In Splatter method, the dropout rate is mainly controlled by two parameters, i.e. “mid” and “shape.” We fix “shape” parameter at −0.5 and set “mid” as 3, 4, and 5 to generate three datasets, in which 65%, 74%, and 81% of the entries are dropouts, and the details are shown in Section 3 of Supplementary Material. Each simulated dataset contains 2000 genes and 500 cells, consisting of five subpopulations. We first calculate the gene-wise and cell-wise Pearson correlation between the reference data and imputed data, then we use root mean-squared error (RMSE) and mean absolute error (MAE) to measure the imputation accuracy of different methods. Besides, as the information of ground truth zeros and dropouts are known in simulated data, we are able to calculate sensitivity and specificity of different methods, measuring the proportion of imputed dropouts and preserved real zeros (Supplementary Table S3). As can be seen, methods including DrImpute, MAGIC, scMOO, and SAVER impute all zeros in the expression matrix, leading to high risk of over-imputation. In contrast, scRMD perfectly avoids over-imputation with 100% of true zeros unchanged, at the cost of failing to identify most dropouts. Among all eight methods compared, only tsImpute is able to achieve relatively balanced performance.

**Figure 2.**
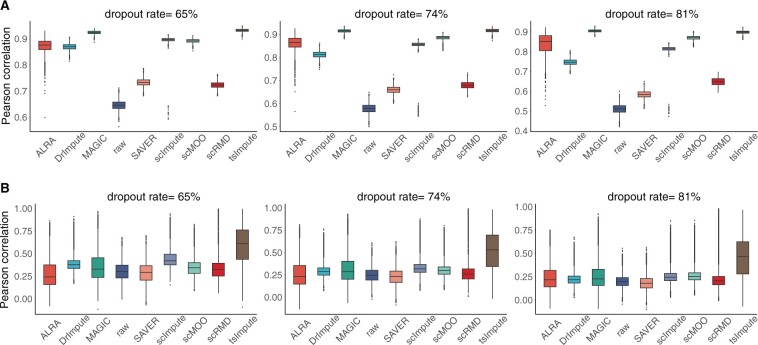
Pearson correlation between the real and imputed values of the simulated data under different dropout rates. Higher correlation coefficients indicate better imputation performance. (A) Cell-wise correlation. (B) Gene-wise correlation.

The cell-wise and gene-wise Pearson correlation are shown in Fig. 2. It can be seen that all imputation methods are able to improve the cell-wise correlation to different extent (Fig. 2A), while ALRA and SAVER fail to recover the gene-wise correlation (Fig. 2B). Among these methods, tsImpute and MAGIC achieve the highest cell-wise correlation, and tsImpute outperforms all other methods in terms of gene-wise correlation. The correlation results within different cell types of the data are shown in Supplementary Fig. S1. Similar to the results of overall cell-wise correlation, all imputation methods considered in our article are able to improve cell-wise correlation within all five cell types of simulated data, among which tsImpute and MAGIC still achieved the best performance. As for gene-wise correlation, tsImpute is still the only method that can consistently enhance gene-wise correlation within different cell types. Besides, to investigate whether tsImpute is able to recover the gene co-expression patterns of the data, we adopted the R package ESCO (Tian *et al.* 2021) to visualize the gene–gene correlation of ground truth expression, observed expression, and imputed results of tsImpute (Supplementary Fig. S2). As can be seen, tsImpute is able to recover the co-expression patterns even when most entries of the data are dropouts. We then compare the imputation accuracy with MAE and RMSE (Supplementary Fig. S3). In all three cases, tsImpute consistently achieves the lowest RMSE and MAE, indicating that tsImpute provides highest imputation accuracy. Overall, tsImpute is able to impute the simulated data and recover its information.

### 3.2 Imputation accuracy in real data

**Figure 3.**
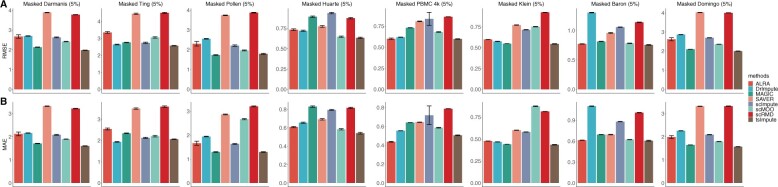
Imputation accuracy on eight real datasets measured by RMSE and MAE, lower RMSE, and MAE indicate better performance. (A) RMSE between the imputed values and real values. (B) MAE between the imputed values and real values.

After testing the imputation performance on simulated data, we now evaluate the imputation accuracy with several real datasets. Datasets used for comparison include four small datasets, i.e. Ting (Ting *et al.* 2014), Darmanis (Darmanis *et al.* 2015) Pollen (Pollen *et al.* 2014), and Huarte(Uriarte Huarte *et al.* 2021) data, which contain less than 500 cells, and four large datasets, namely PBMC (Zheng *et al.* 2017), Klein (Klein *et al.* 2015), Baron (Baron *et al.* 2016), and Domingo data (Domingo-Gonzalez *et al.* 2020), which contain approximately 3000–4000 cells, and a more detailed description of these datasets is shown in Supplementary Table S1. Specifically, we randomly mask 5% and 10% non-zero entries of these data to generate artificial dropouts, then all imputation methods are used to recover the masked data. After imputing the masked data, we calculate RMSE and MAE between the imputed data and ground true values of the masked data, which is similar to the simulation study. However, as the information of true zeros in real data is unavailable, the calculation of correlation coefficient will be biased, hence we do not calculate the cell-wise or gene-wise correlation. Each imputation method is implemented for 10 times. Figure 3 shows the results of different imputation methods on all datasets with 5% artificially masked values. It can be seen that tsImpute achieves lowest RMSE in seven of eight datasets, the only exception is Pollen data, in which MAGIC generates the lowest RMSE and tsImpute takes the second place. In terms of MAE, tsImpute outperforms its competitors in six of eight datasets, while DrImpute and ALRA generate the lowest MAE in Ting and PBMC data, respectively, followed by tsImpute. The results with masking rate equal to 10% are shown in Supplementary Fig. S4. As more non-zero entries are masked, the imputation accuracy of all methods declines. Still, tsImpute outperforms other methods in six of eight datasets in terms of both MAE and RMSE, and only MAGIC is able to outperform tsImpute in Pollen and Klein data.

### 3.3 TsImpute improves identification of cell subpopulations

**Figure 4.**
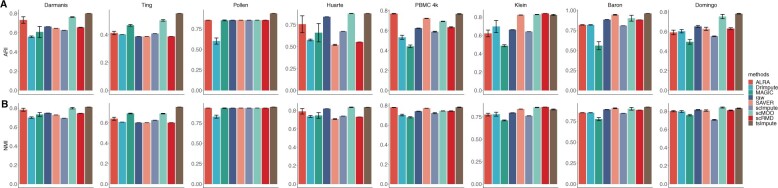
Clustering results of raw data and different imputation methods on eight real datasets. The performance is measured by ARI and NMI, higher ARI and NMI mean better clustering performance. (A) ARI scores of different methods. (B) NMI scores of different methods.

We now investigate the performance of different imputation methods for clustering analysis. We use the same eight datasets as in Section 3.2 for evaluation. Before imputation starts, 2000 highly variable genes are selected with Seurat (Butler *et al.* 2018) package and all imputation methods are performed on the filtered data. We adopt the shared nearest neighbor (Waltman and van Eck 2013) based method for cell clustering, which is also the default clustering method of Seurat package. The first 10 principal components of the data are used for clustering. Adjusted Rand index (ARI) (Hubert and Arabie 1985) and normalized mutual information (NMI) (Strehl and Ghosh 2002) are used to evaluate the clustering results, of which larger values indicate better clustering results. Each method is run for 10 times to avoid the influence of stochasticity. Figure 4 illustrates the clustering results. As can be seen, in Darmanis, Ting, Pollen, Huarte, Baron, and Domingo datasets, tsImpute achieves the highest ARI among all eight imputation methods. In PBMC data, tsImpute takes the second place, while in Klein data scRMD and scMOO have higher ARI than tsImpute. In terms of NMI, the performance of tsImpute is similar to that of ARI: tsImpute takes the lead in four of eight datasets and has the second-best NMI in PBMC, Huarte, and Domingo data. In Klein data, scRMD, SAVER, and scMOO generates higher NMI than tsImpute. Nevertheless, tsImpute outperforms its competitors in most cases, and it is the only method that consistently improves the clustering performance compared to raw data in all eight datasets. Overall, tsImpute effectively improves identification of cell subpopulations, which indicates that tsImpute accurately imputes the dropouts and recovers the biological information.

### 3.4 TsImpute improves differential expression analysis

DE analysis is one of the main downstream tasks of scRNA-seq analysis, which aims to define the sets of genes that best discriminate different subpopulations of cells. However, the prevalence of dropouts in scRNA-seq data will deteriorate the performance of DE analysis. In this section, we evaluate the efficacy of imputation methods by comparing the performance between DE analysis on raw data and imputed data. We consider the Chu data (Chu *et al.* 2016), which is a human embryonic stem cell dataset consisting of both scNRA-seq data and bulk RNA-seq data. We construct two datasets from the single-cell Chu data, one contains 138 definitive endoderm cells (DEC) and 212 human embryonic stem cells (H1 ESC), while the other consists of 105 endothelial cells (EC) and 212 H1 ESC cells. As bulk RNA-seq data is less likely to be influenced by dropouts, we use the DE genes identified from bulk Chu data as the reference.

**Figure 5.**
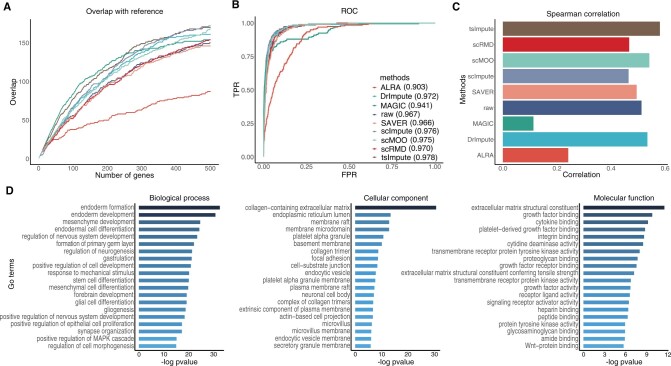
DE analysis results of raw data and different imputation methods. (A) Overlap of the single-cell DE genes and reference bulk DE genes. (B) ROC curves and corresponding AUC values of different imputation methods. (C) Spearman correlation between adjusted *P* values derived from bulk data and single cell data. (D) Statistically significant GO terms identified with ClusterProfiler, which are divided into three categories, i.e. biological process, cellular component, and molecular function.

We first consider the dataset comprised H1 and DEC cells. The popular R package edgeR (Robinson *et al.* 2009) is used to identify DE genes. With the maximum false discovery rate of 0.01 and minimum log fold-change of 2, edgeR identified 776 DE genes from the bulk data. We choose the top 200 genes ranked by adjusted *P* values as the gold standard reference, then compare them with the genes detected from raw and imputed single-cell data to evaluate the performance of different methods. Figure 5A shows the overlap between reference DE genes generated from bulk data and those identified from raw and imputed data. It can be seen that scImpute, tsImpute, and DrImpute are the top 3 methods that identify the most gold standard DE genes, while SAVER and ALRA do not improve the performance of DE analysis over raw data. We then draw the receiver operating characteristic (ROC) curves and calculate the corresponding area under curve (AUC) values of different methods. As can be seen in Fig. 5B, tsImpute achieves the highest AUC score, followed by scImpute and scMOO, indicating that these three methods are most likely to assign significant adjusted *P* values to ground true DE genes, while ALRA, MAGIC, and SAVER do not generate higher AUC scores than that of raw data. Figure 5C displays the Spearman correlation coefficients between adjusted *P* values generated from bulk data and imputed (raw) data, and the results indicate that DE genes identified by tsImpute are the most consistent with the gold standard DE genes identified from bulk data. To further investigate whether tsImpute can retain and recover the biological information of the scRNA-seq data, we conduct gene ontology (GO) term enrichment analysis on the 170 overlapped genes identified by both tsImpute and the bulk data. The R package clusterProfiler (Wu *et al.* 2021) is used to generate the GO enrichment analysis results in Fig. 5D. It can be observed that the most enriched GO terms are highly relative to the differentiation from embryonic cells to DEC, such as endoderm formation (GO:0001706), endoderm development (GO:0007492), endodermal cell differentiation (GO:0035987), etc. The results of GO term enrichment analysis are consistent with the description of the datasets, indicating that tsImpute can effectively impute dropouts while preserving the biological information of the data. We then focus on the second dataset consisting of DEC and EC cells. As is shown in Supplementary Fig. S5, tsImpute outperforms all other competitors in terms of overlap with reference DE genes, AUC scores, and Spearman correlation. Besides, the GO terms derived from DE genes identified by tsImpute also reflect the biological information of the dataset (Supplementary Fig. S6), e.g. EC migration (GO:0043542), endothelial development (GO:0003158), and so on.

### 3.5 Ablation study

As is mentioned above, tsImpute consists of two steps of imputation, i.e. ZINB imputation and IDW imputation. Besides, before the initial ZINB imputation, tsImpute adopts Jaccard clustering based on highly expressed genes. To validate the significance of these components, in this section, we perform ablation tests on all real datasets used in clustering analysis. We remove ZINB imputation, IDW imputation, and replace the Jaccard clustering with Seurat clustering respectively, then use these ablated models for cell clustering. ARI and NMI are used as evaluation metrics and each experiment is repeated for 10 times. The numerical results of the ablation tests measured by ARI are shown in Table 1 and the results of NMI are shown in Supplementary Table S4. It can be seen that in most cases removing or altering any part of tsImpute deteriorates its performance. Besides, removing ZINB imputation drastically declines the clustering performance, indicating that directly conducting imputation on raw expression data with dropouts may introduce extra noises and hence impair the imputation performance. It is noteworthy that retaining ZINB imputation while removing IDW imputation has relatively less influence on the clustering results, in some cases it may even generate better results than the complete model, which further demonstrates the efficacy of ZINB imputation. Still, only the complete model can consistently improve clustering results over raw data, which validates the significance of tsImpute. In addition, to test whether Jaccard clustering is robust to changes of clustering method, we replace hierarchical clustering with partitioning around medoids clustering method and compare their clustering performance. Results are shown in Supplementary Table S5. It can be seen that replacing clustering method in the Jaccard clustering step does not significantly decrease the performance of tsImpute, indicating that tsImpute is robust to the changes of clustering methods in Jaccard clustering step.

**Table 1.** Results of ablation test.^a^

Jaccard clustering	ZINB imputation	IDW imputation	Pollen	Ting	Darmanis	Huarte	Klein	Baron	PBMC	Domingo
✓	✓	✓	**0.938**	**0.553**	**0.800**	**0.879**	0.822	**0.962**	**0.767**	**0.782**
✓	✓	✗	0.900	0.500	0.769	0.853	**0.827**	0.819	0.521	0.722
✓	✗	✓	0.848	0.500	0.464	0.576	0.168	0.201	0.147	0.210
✗	✓	✓	0.856	0.386	0.554	0.549	0.560	0.510	0.481	0.575
✗	✓	✗	0.856	0.388	0.556	0.549	0.562	0.590	0.647	0.578
✗	✗	✓	0.848	0.432	0.355	0.464	0.161	0.201	0.128	0.260
✗	✗	✗	0.856	0.388	0.663	0.842	0.662	0.884	0.624	0.653

a TsImpue is compared to ablated models which remove Jaccard clustering, ZINB imputation, or IDW imputation, and the last line of the table denotes clustering results of raw data. ARI is used as evaluation metric. Best results are marked in bold.

## 4 Conclusion

The prevalence of dropouts is one of the major issues in scRNA-seq data analysis. In this article, we propose a novel method tsImpute to address the challenge of dropouts in a two-step manner. Lots of existing imputation methods rely on cell clustering and involve calculating distance based on the raw expression matrix, which tends to be distorted due to the numerous dropouts. Besides, most imputation methods impute all zeros in the expression matrix, which may also introduce extra noise into the data. To overcome these drawbacks, tsImpute first identifies likely dropouts with ZINB distribution and calculates initial imputed values combining information from both cells and genes. After initial imputation, tsImpute adopts IDW method to conduct final imputation only on the dropouts identified in the first step to avoid over-imputation. To assess the performance of tsImpute, extensive studies including masking experiments on both simulated and real data, clustering analysis and DE analysis are conducted, and tsImpute is compared with several state-of-the-art imputation methods. The numerical results show that tsImpute achieves desirable performance. Besides, as tsImpute is a two-step method, we also conduct ablation studies to validate the significance of each step, results of which prove the necessity of each component contained in tsImpute.

It is noteworthy that although ZINB distribution is widely used in modeling the expression level of scRNA-seq data, there are also studies adopting other distributions for data imputation, hence the performance of tsImpute may be further improved if a more appropriate distribution of scRNA-seq data is found. Besides, although tsImpute is able to handle thousands of cells in a few minutes, methods involving distance matrix calculation such as tsImpute, scImpute, and DrImpute are not as fast as methods based on matrix decomposition (Supplementary Table S2), and their computation speed may be further improved with parallel computation methods. Furthermore, to avoid over-imputation, tsImpute fills only the zeros identified as dropouts by ZINB distribution and does not alter the non-zero values. However, the non-zero entries may not represent the true expression levels, hence the expression matrix can be further denoised by properly modifying those non-zero values.

## Supplementary Material

btad731_Supplementary_Data

## Data Availability

The datasets underlying this article are available at https://github.com/ZhengWeihuaYNU/tsImpute.
