# Supplementary material for: TsImpute: an accurate two-step imputation method for single-cell RNA-seq data
Source: Bioinformatics. 2023 Dec 1;39(12):btad731. doi: 10.1093/bioinformatics/btad731 (PMC10724850; doi:10.1093/bioinformatics/btad731)
Supplement: btad731_Supplementary_Data [file btad731_supplementary_data.pdf]

Supplementary data to 'TsImpute: An accurate two-step imputation method for single-cell RNA-seq data'

Weihua Zheng, Wenwen Min and Shunfang Wang

November 22, 2023

1 Data preprocessing

TsImpute takes as input a raw count matrix $X_{m\times n}$ with m genes and n cells. To find highly variable genes, we first generate the normalized expression matrix $\mathbf{Y} = (y_{ij})_{m\times n}$, which is defined as

$$y_{ij} = \log_{10}(\frac{x_{ij}}{\sum_{k=1}^{m} x_{kj}} \times 1000000 + 1), i = 1, ..., m, j = 1, ..., n. \quad (1)$$

Then we calculate the coefficient of variance (CV) of each normalized gene:

$$CV(Y_i) = \frac{sd(Y_i)}{\bar{Y}_i}, i = 1, ..., m, \quad (2)$$

in which $sd(Y_i)$ means the standard deviation of Y_i. Then, the 2000 genes with highest CV are passed to next step and others genes will not be imputed. Note that in the subsequent step tsImpute still uses the raw counts of these highly variable genes as ZINB model requires count data as input.

2 Description of datasets

In this article, nine real datasets are used for evaluation, i.e. Darmanis (Darmanis *et al.*, 2015), Ting (Ting *et al.*, 2014), Pollen (Pollen *et al.*, 2014), Huarte (Uriarte Huarte *et al.*, 2021), PBMC (Zheng *et al.*, 2017), Klein (Klein *et al.*, 2015), Baron (Baron *et al.*, 2016), Domingo(Domingo-Gonzalez *et al.*, 2020) and Chu (Chu *et al.*, 2016). The description and availability of these data are displayed as below:

Table S1: Description and availability of datasets used in the article.

Data	Genes	Cells	Groups	Source
Darmanis	22083	466	9	GSE67835
Ting	21651	187	7	GSE51372
Pollen	23794	299	11	SRP041736
Huarte	22672	367	8	GSE148393
PBMC	16653	4271	8	10X Genomics
Klein	24175	2717	4	GSE65525
Baron	20125	3605	14	GSE84133
Domingo	18072	4052	15	GSE147668
Chu	19097	1018	6	GSE75748

3 Generation of simulated data

The simulated datasets used in the article are generated using the R package Splatter (Zappia *et al.*, 2017). More specifically, the datasets can be generated with the following R codes:

```
BiocManager::install('splatter')
library(splatter)
rate<- -0.5 #fix this parameter
mid<- 5 #set this as 3, 4 or 5
params = newSplatParams()
params = setParams(params, list(batchCells = 500,
nGenes =2000,
group.prob = rep(0.2, 5),
de.prob = c(0.05, 0.08, 0.01, 0.1, 0.1),
de.facLoc = 0.5,
de.facScale = 0.8,
seed= 1))

# Generate the simulation data using Splatter package
sim = splatSimulateGroups(params,
dropout.shape =rep(rate, 5),
dropout.mid = rep(mid,5),
dropout.type = "group")
counts     <- as.data.frame(counts(sim)) #observed count matrix
truecounts <- as.data.frame(assays(sim)$TrueCounts) #true count matrix
dropout    <- as.matrix(assays(sim)$Dropout) #dropout matrix
```

4 Methods for comparison

In this article, seven imputation methods are used for comparison, namely scImpute (Li and Li, 2018), DrImpute (Gong *et al.*, 2018), MAGIC (Dijk *et al.*, 2018), scRMD (Chen *et al.*, 2020), ALRA (Linderman *et al.*, 2022), SAVER (Huang *et al.*, 2018) and scMOO (Jin *et al.*, 2022). The availability of these methods is as follows:

4.1 scImpute

The R package of ScImpute can be downloaded from `https://github.com/Vivianstats/scImpute` and it is implemented with default parameters.

4.2 DrImpute

We download the R package of DrImpute from `https://github.com/gongx030/DrImpute` and implement it with default parameters.

4.3 MAGIC

The R package of MAGIC is aquired from `https://github.com/KrishnaswamyLab/MAGIC` , and the algorithm is implemented with deault parameters.

4.4 scRMD

The R package of scRMD is downloaded from `https://github.com/XiDsLab/scRMD`, and we implement it with default parameters.

4.5 ALRA

The R source codes of ALRA can be aquired from `https://github.com/KlugerLab/ALRA`, we run the source codes with default parameters.

4.6 SAVER

The R package of SAVER can be downloaed from `https://github.com/mohuangx/SAVER` and it is implemented with default parameters.

4.7 scMOO

The R package of scMOO can be aquired from `https://github.com/Zhangxf-ccnu/scMOO` and we implement it with default parameters.

5 Evaluation metrics

In this article, several evaluation metrics are used to assess the performance of different imputation methods, in this section we list the formulae of these metrics, including rooted mean squared error (RMSE), mean absolute error (MAE), Spearman correlation coefficient, Pearson correlation coefficient, sensitivity, specificity, adjusted Rand Index (ARI) (Hubert and Arabie, 1985) and normalized mutual information (NMI) (Strehl and Ghosh, 2002).

5.1 RMSE and MAE

Suppose $X = (x_1, ..., x_n)$ is the true value and $\hat{X} = (\hat{x_1}, ..., \hat{x_n})$ is the predicted value, RMSE and MAE can be calculated by

$$RMSE = \sqrt{\frac{\sum_{i=1}^{n}(x_i - \hat{x_i})^2}{n}} \tag{3}$$

and

$$MAE = \frac{|\sum_{i=1}^{n}(x_i - \hat{x_i})|}{n}. \tag{4}$$

5.2 Pearson correlation and Spearman correlation

Suppose $X = (x_1, ..., x_n)$ is the true value and $Y = (y_1, ..., y_n)$ is the predicted value, Pearson correlation coefficient can be calculated by

$$\rho_p = \frac{\sum_{i=1}^{n}(x_i - \bar{x})(y_i - \bar{y})}{\sqrt{\sum_{i=1}^{n}(x_i - \bar{x})^2 \sum_{i=1}^{n}(y_i - \bar{y})^2}}. \tag{5}$$

As for Spearman correlation, it is defined as

$$\rho_s = 1 - \frac{6\sum d_i^2}{n(n^2 - 1)}, \tag{6}$$

where d_i is the rank difference between x_i and y_i.

5.3 Sensitivity, specificity and balanced accuracy

Consider the confusion matrix of imputation:

	Imputed	Not imputed
Dropouts	TP	FN
True zeros	FP	TN

Sensitivity, specificity and balanced accuracy can be calculated as follows:

$$Sensitivity = \frac{TP}{TP + FN}, \tag{7}$$

$$Specificity = \frac{TN}{FP + TN}, \tag{8}$$

$$Balanced\ accuracy = \frac{sensitivity + specificity}{2}. \tag{9}$$

5.4 ARI and NMI

ARI and NMI are two popular evaluation metrics which assess the performance of clustering analysis, ARI is defined as

$$ARI(A^*, A) = \frac{\sum_{i,j} \binom{N_{ij}}{2} - [\sum_i \binom{N_i}{2} \sum_j \binom{N_j}{2}]/\binom{N}{2}}{\frac{1}{2}[\sum_i \binom{N_i}{2} + \sum_j \binom{N_j}{2}] - [\sum_i \binom{N_i}{2} \sum_j \binom{N_j}{2}]/\binom{N}{2}}, \tag{10}$$

where N is the number of cells, N_{ij} is the number of cells of the real cell type $C_j^* \in A^*$ assgined to cluster C_i in partition A, and N_j is the number of cells of cell type C_j^*. The value range of ARI is $[-1, 1]$, a higher ARI indicates better clustering results, and ARI equals to 1 only when the clustering result is identical to the real cell type partition. NMI is defined as follows:

$$NMI(A^*, A) = \frac{2 \times I(A^*, A)}{H(A^*) + H(A)}, \tag{11}$$

in which $I(A^*, A) = H(A^*) - H(A^*|A)$, $H(A) = -\sum_{a \in A} p(a) \log_2(p(a))$, $H(A^*|A) = H(A^*, A) - H(A)$ and $H(A^*, A) = -\sum_{a^*, a} p(a^*, a) \log_2(p(a^*, a))$. The value of NMI falls between $[0, 1]$ and a larger NMI means better clustering performance.

5.5 Silhouette coefficient

Silhouette coefficient is a metric that can be used to evaluate clustering results without knowing the ground truth type labels. For a given cell i, silhouette coefficient can be calculated as follows:

$$S(i) = \frac{b(i) - a(i)}{max\{a(i), b(i)\}}, \tag{12}$$

where $a(i)$ is the average distance between cell i and other cells of the same cluster, and $b(i)$ denotes the average distance between cell i and its nearest cluster. The overall result is generated by averaging silhouette coefficients of all cells. The value of silhouette coefficient is between -1 and 1, and a larger value indicates better clustering performance.

6 Pseudo-codes of EM algorithm for ZINB paramters estimation

In this article, the parameters of ZINB distribution are estimated with expectation maximization (EM) algorithm (Dempster *et al.*, 1977). EM algorithm optimizes the parameters in a iterative manner:

Algorithm 1 EM algorithm for ZINB parameters estimation

Input: a gene vector $g = (x_1, ..., x_m)$
Process:
Initialize the ZINB parameters $\pi = \pi_0, r = r_0$ and $p = p_0$, maximum number of iterations t_{max}, minimum difference ϵ, $t = 1$, $lik = 0$, $diff = 100$
while $t \leq t_{max}$ and $diff \geq \epsilon$ **do**
  E step: calculate $\pi_t = \frac{\pi_{t-1}}{\pi_{t-1}+(1-\pi_{t-1}p^r)}$
  M step: estimate p_t and r_t by maximizing $l_{ZINB} = \sum_{i=1}^{m}(1 - \pi_t)[\log \frac{(x_i+r-1)!}{x_i!(r-1)!} - r\log(\frac{1-p}{p}) + x_i \log(1-p)]$
  Calculate the log-likelihood $L(\pi_t, r_t, p_t)$ with current parameters, update $diff$ and lik by $diff = |L(\pi_t, r_t, p_t) - lik|$ and $lik = L(\pi_t, r_t, p_t)$
  Update: $\pi = \pi_t, r = r_t, p = p_t$
  t= t+1
end while
return π, r, p

7 Pseudo-codes of tsImpute

Algorithm 2 Pseudo-codes of tsImpute

Input: expression matrix $X_{m \cdot n}$, number of top genes n_{top} and dropout threshold $thres$
Process:
Binarize each cell vector $x_i (i = 1, ..., n)$ by converting the n_{top} highest expressed genes to 1 and others to 0
Decide the optimal number of clusters k according silhouette coefficient
Divide cells into k clusters with Jaccard distance, in which cluster i includes n_i cells
Step 1: ZINB imputation:
for i in 1:k **do**
  for j in 1:m **do**
    Estimate ZINB parameters π_j^i, r_j^i, p_j^i for gene j in cells of cluster i
  end for
  for l in 1:n_i **do**
    Calculate scale factor $s_l^i = n_i \cdot \sum_{j=1}^{m} x_{jl}^k / \sum_j \sum_l x_{jl}$
    for j in 1:m **do**
      Calculate imputed values of ZINB imputation by $x_{jl}^{init} = \begin{cases} \frac{n_i \cdot \pi_j^i}{\sum_l I(x_{jl}=0)} \cdot \pi_j^i \cdot \frac{r_j^i(1-p_j^i)}{p_j^i} \cdot s_l^i, & if\ \frac{n_i \cdot \pi_j^i}{\sum_l I(x_{jl}=0)} \geq thres \\ x_{jl}, & otherwise. \end{cases}$
    end for
  end for
end for
Step 2: IDW imputation:
Given the ZINB imputed expression matrix X^{init}, normalize the expression levels to generate Y
Divide cells into C clusters, where cluster c includes n_c cells
for i in 1:C **do**
  Calculate the Euclidean distance matrix D^i according to Y^i
  Calculate the inverse distance matrix W^i
  for $j = 1 : m$ **do**
    for $k = 1 : n_c$ **do**
      Calculate the final imputed value $y_{jk}^{final} = \begin{cases} \sum_{k=1}^{n_c} w_{jk} y_{jk}, & if\ y_{jk}\ is\ a\ dropout \\ y_{jk}, & otherwise. \end{cases}$
    end for
  end for
end for
return The final imputed expression matrix Y^{final}

8 Time cost of different methods

In this section, we display the time consumption of different methods on eight real data used in data masking experiment. All methods are implemented on Apple MacBook Pro M1 2020 (3.2 GHz Apple M1 8-core CPU and 16-GB RAM) for ten times, and the time cost table is shown as below (average time cost $\pm$ standard deviation):

Table S2: Time consumption of different methods on real datasets.

	Data							
	Pollen	Ting	Darmanis	Huarte	Klein	Baron	PBMC	Domingo
ALRA	1.45±0.25s	1.13±0.19s	1.87±0.08s	1.28±0.09s	11.10±0.37s	13.71±0.47s	16.59±0.65s	12.76±0.67s
DrImpute	42.77±0.33s	17.38±0.46s	105.62±0.65s	9.08±0.08s	>3600s	>3600s	>3600s	>3600s
MAGIC	0.16±0.03s	3.57±0.09s	0.55±0.99s	4.75±0.45s	2.07±0.14s	3.60±0.07s	5.22±0.15s	11.06±0.41s
SAVER	92.40±2.44s	53.78±0.79s	130.21±2.70s	25.36±0.76s	354.29±30.43s	449.29±13.05s	322.75±13.03s	437.05±27.82s
scImpute	29.45±0.56s	22.89±1.61s	34.53±0.61s	13.13±0.39s	877.43±37.42s	1492.50±61.63s	2198.22±94.17s	1332.81±19.33s
SCMOO	166.73±7.39s	165.92±0.63s	169.27±6.81s	85.02±1.89s	191.36±0.70s	297.15±44.20s	266.02±6.93s	593.11±31.33s
scRMD	2.64±1.99s	1.15±0.46s	3.62±0.47s	1.87±0.04s	23.67±0.32s	26.55±0.53s	33.37±1.52s	67.29±0.65s
tsImpute	88.91±1.53s	37.87±0.64s	75.01±1.25s	37.05±0.98s	300.65±18.66s	535.73±12.22s	535.03±22.98s	460.33±26.04s

9 Supplementary tables and figures

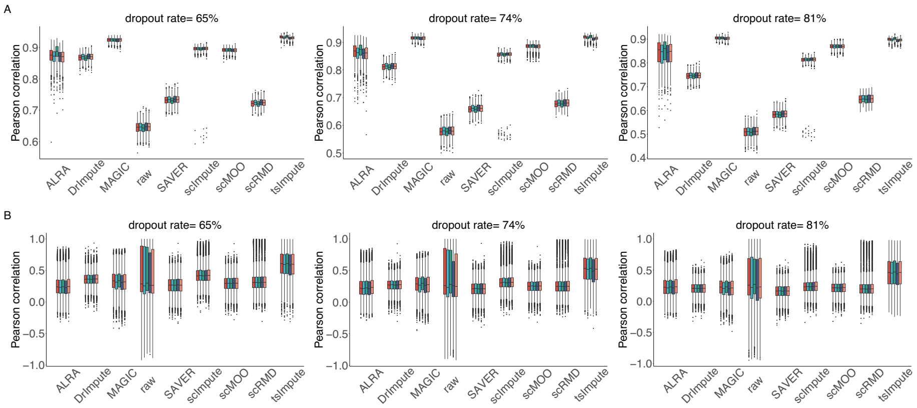

Figure S1: Cell-wise and gene-wise Pearson correlation between the real and imputed values within different cell types of the simulated data. Higher correlation coefficients indicate better imputation performance.(A) Cell-wise correlation. (B) Gene-wise correlation.

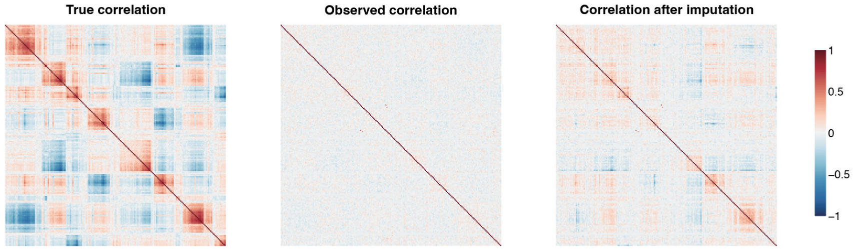

Figure S2: Heatmap of gene-gene correlation with 81% dropouts in the simulated data. Left: correlation of true expression. Middle: correlation of observed expression with dropouts. Right: correlation recoverd by tsImpute.

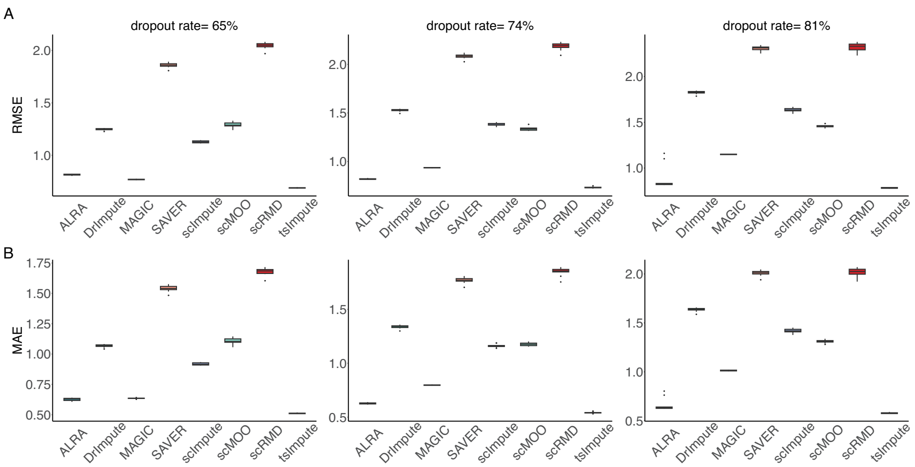

Figure S3: Imputation accuracy on simulated datasets measured by RMSE and MAE. Lower RMSE and MAE indicate better imputation performance. (A) RMSE between the real values and imputed values generated by different imputation methods. (B) MAE between the real values and imputed values generated by different imputation methods.

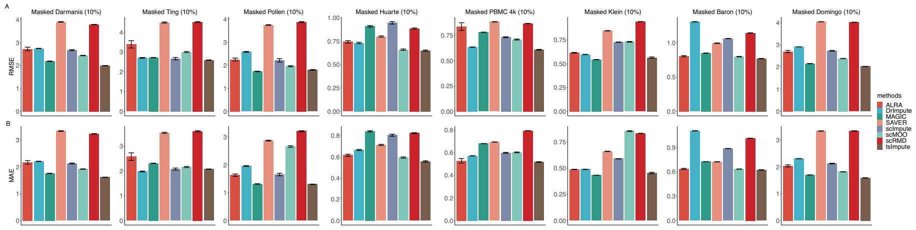

Figure S4: Imputation accuracy on eight real datasets measured by RMSE and MAE, lower RMSE and MAE indicate better performance. (A) RMSE between the imputed values and real values. (B) MAE between the imputed values and real values.

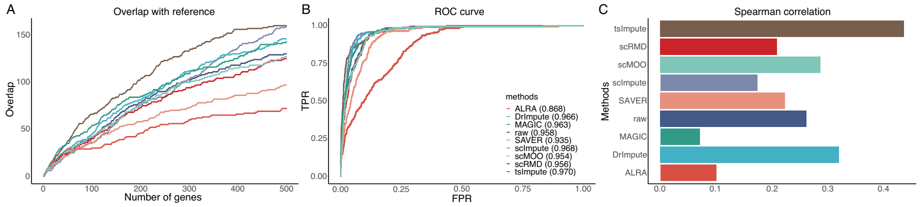

Figure S5: DE analysis results of H1 ESC versus EC data. (A) Overlap of single-cell DE genes with reference DE genes. (B) ROC curves and AUC values of different imputation methods. (C) Spearman correlation between adjusted P values derived from bulk data and single cell data.

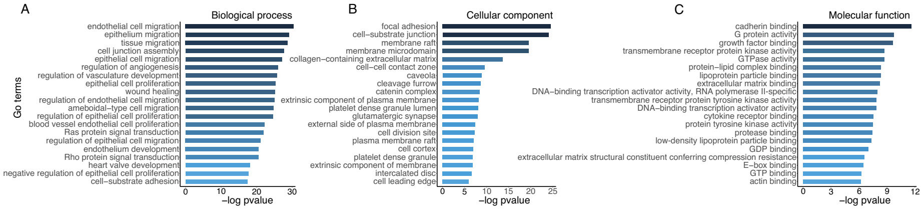

Figure S6: Statistically significant GO terms identified with ClusterProfiler, which are divided into three categories, i.e. biological process, cellular component and molecular function.

Table S3: Sensitivity, specificity and balanced accuracy of all methods on simulated data.

Metrics	Dropout rate	tsImpute	scImpute	DrImpute	MAGIC	ALRA	scRMD	scMOO	SAVER
Sensitivity	65%	90.5%	99.7%	100%	100%	95.8%	3.3%	100%	100%
	74%	89.5%	99.1%	100%	100%	96.4%	3.1%	100%	100%
	81%	88.1%	99.0%	100%	100%	94.1%	3.3%	100%	100%
Specificity	65%	35.8%	1.6%	0%	0%	20.6%	100%	0%	0%
	74%	38.7%	2.9%	0%	0%	15.9%	100%	0%	0%
	81%	41.5%	4.3%	0%	0%	17.6%	100%	0%	0%
Balanced Accuracy	65%	63.2%	50.7%	50.0%	50.0%	58.2%	51.7%	50.0%	50.0%
	74%	64.1%	51.0%	50.0%	50.0%	56.2%	51.6%	50.0%	50.0%
	81%	64.8%	51.7%	50.0%	50.0%	55.9%	51.7%	50.0%	50.0%

Table S4: Results of ablation test measured by NMI. Best results are marked in bold.

Jaccard clustering	ZINB imputation	IDW imputation	Pollen	Ting	Darmanis	Huarte	Klein	Baron	PBMC	Domingo
✓	✓	✓	**0.948**	**0.754**	0.817	**0.836**	0.832	**0.920**	**0.781**	**0.833**
✓	✓	×	0.927	0.672	**0.819**	0.816	**0.838**	0.850	0.712	0.815
✓	×	✓	0.925	0.704	0.707	0.751	0.511	0.575	0.532	0.545
×	✓	✓	0.935	0.602	0.719	0.730	0.750	0.758	0.690	0.781
×	✓	×	0.935	0.601	0.722	0.730	0.752	0.794	0.756	0.788
×	×	✓	0.925	0.704	0.565	0.671	0.502	0.575	0.515	0.649
×	×	×	0.935	0.601	0.749	0.820	0.795	0.890	0.740	0.817

Table S5: Robustness test of Jaccard clustering. ARI and NMI are used as evaluation metrics.

		Pollen	Ting	Darmanis	Huarte	Klein	Baron	PBMC	Domingo
ARI	Hierarchical	0.938	0.553	0.800	0.879	0.822	0.962	0.767	0.782
	PAM	0.938	0.546	0.774	0.858	0.810	0.956	0.780	0.761
	raw	0.856	0.388	0.663	0.842	0.662	0.884	0.624	0.653
NMI	Hierarchical	0.948	0.754	0.817	0.836	0.832	0.920	0.781	0.833
	PAM	0.948	0.744	0.779	0.820	0.830	0.910	0.780	0.813
	raw	0.935	0.601	0.749	0.820	0.795	0.890	0.740	0.817

References

Baron, M., Veres, A., Wolock, S. L., Faust, A. L., Gaujoux, R., Vetere, A., Ryu, J. H., Wagner, B. K., Shen-Orr, S. S., Klein, A. M., Melton, D. A., and Yanai, I. (2016). A single-cell transcriptomic map of the human and mouse pancreas reveals inter- and intra-cell population structure. *Cell Systems*, **3**(4), 346–360.e4.

Chen, C., Wu, C., Wu, L., Wang, X., Deng, M., and Xi, R. (2020). scRMD: imputation for single cell RNA-seq data via robust matrix decomposition. *Bioinformatics*, **36**(10), 3156–3161.

Chu, L. F., Leng, N., Zhang, J., Hou, Z., Mamott, D., Vereide, D. T., Choi, J., Kendziorski, C., Stewart, R., and Thomson, J. A. (2016). Single-cell rna-seq reveals novel regulators of human embryonic stem cell differentiation to definitive endoderm. *Genome Biology*, **17**(1), 173.

Darmanis, S., Sloan, S. A., Zhang, Y., Enge, M., Caneda, C., Shuer, L. M., Gephart, M. G. H., Barres, B. A., and Quake, S. R. (2015). A survey of human brain transcriptome diversity at the single cell level. *Proceedings of the National Academy of Sciences*, **112**(23), 7285–7290.

Dempster, A., Laird, N., and Rubin, D. (1977). Maximum likelihood from incomplete data via the em algorithm. *Journal of the Royal Statistical Society. Series B (Methodological)*, **39**, 1–38.

Dijk, D., Sharma, R., Nainys, J., Yim, K., Kathail, P., Carr, A., Burdziak, C., Moon, K., Chaffer, C., Pattabiraman, D., Bierie, B., Mazutis, L., Wolf, G., Krishnaswamy, S., and Pe'er, D. (2018). Recovering gene interactions from single-cell data using data diffusion. *Cell*, **174**.

Domingo-Gonzalez, R., Zanini, F., Che, X., Liu, M., Jones, R. C., Swift, M. A., Quake, S. R., Cornfield, D. N., and Alvira, C. M. (2020). Diverse homeostatic and immunomodulatory roles of immune cells in the developing mouse lung at single cell resolution. *eLife*, **9**, e56890.

Gong, W., Kwak, I.-Y., Pota, P., Koyano-Nakagawa, N., and Garry, D. J. (2018). Drimpute: imputing dropout events in single cell rna sequencing data. *BMC Bioinformatics*, **19**(1), 220.

Huang, M., Wang, J., Torre, E., Dueck, H., Shaffer, S., Bonasio, R., Murray, J. I., Raj, A., Li, M., and Zhang, N. R. (2018). Saver: gene expression recovery for single-cell rna sequencing. *Nature Methods*, **15**(7), 539–542.

Hubert, L. and Arabie, P. (1985). Comparing partitions. *J. Classif*, **2**(1), 193–218.

Jin, K., Li, B., Yan, H., and Zhang, X.-F. (2022). Imputing dropouts for single-cell RNA sequencing based on multi-objective optimization. *Bioinformatics*, **38**(12), 3222–3230.

Klein, A. M., Mazutis, L., Akartuna, I., Tallapragada, N., Veres, A., Li, V., Peshkin, L., Weitz, D. A., and Kirschner, M. W. (2015). Droplet barcoding for single-cell transcriptomics applied to embryonic stem cells. *Cell*, **161**(5), 1187–1201.

Li, W. V. and Li, J. J. (2018). An accurate and robust imputation method scimpute for single-cell rna-seq data. *Nature Communications*, **9**(1), 997.

Linderman, G. C., Zhao, J., Roulis, M., Bielecki, P., Flavell, R. A., Nadler, B., and Kluger, Y. (2022). Zero-preserving imputation of single-cell rna-seq data. *Nature Communications*, **13**(1), 192.

Pollen, A. A., Nowakowski, T. J., Shuga, J., Wang, X., Leyrat, A. A., Lui, J. H., Li, N., Szpankowski, L., Fowler, B., Chen, P., Ramalingam, N., Sun, G., Thu, M., Norris, M., Lebofsky, R., Toppani, D., Kemp, D. W., Wong, M., Clerkson, B., Jones, B. N., Wu, S., Knutsson, L., Alvarado, B., Wang, J., Weaver, L. S., May, A. P., Jones, R. C., Unger, M. A., Kriegstein, A. R., and West, J. A. A. (2014). Low-coverage single-cell mrna sequencing reveals cellular heterogeneity and activated signaling pathways in developing cerebral cortex. *Nature Biotechnology*, **32**(10), 1053–1058.

Strehl, A. and Ghosh, J. (2002). Cluster ensembles - a knowledge reuse framework for combining multiple partitions. *Journal of Machine Learning Research*, **3**, 583–617.

Ting, D. T., Wittner, B. S., Ligorio, M., Jordan, N. V., Shah, A. M., Miyamoto, D. T., Aceto, N., Bersani, F., Brannigan, B. W., Xega, K., Ciciliano, J. C., Zhu, H., MacKenzie, O. C., Trautwein, J., Arora, K. S., Shahid, M., Ellis, H. L., Qu, N., Bardeesy, N., Rivera, M. N., Deshpande, V., Ferrone, C. R., Kapur, R., Ramaswamy, S., Shioda, T., Toner, M., Maheswaran, S., and Haber, D. A. (2014). Single-cell rna sequencing identifies extracellular matrix gene expression by pancreatic circulating tumor cells. *Cell Reports*, **8**(6), 1905–1918.

Uriarte Huarte, O., Kyriakis, D., Heurtaux, T., Pires Afonso, Y., Grzyb, K., Halder, R., Buttini, M., Skupin, A., Mittelbronn, M., and Michelucci, A. (2021). Single-cell transcriptomics and in situ morphological analyses reveal microglia heterogeneity across the nigrostriatal pathway. *Frontiers in Immunology*, **12**.

Zappia, L., Phipson, B., and Oshlack, A. (2017). Splatter: simulation of single-cell rna sequencing data. *Genome Biology*, **18**(1), 174.

Zheng, G. X. Y., Terry, J. M., Belgrader, P., Ryvkin, P., Bent, Z. W., Wilson, R., Ziraldo, S. B., Wheeler, T. D., McDermott, G. P., Zhu, J., Gregory, M. T., Shuga, J., Montesclaros, L., Underwood, J. G., Masquelier, D. A., Nishimura, S. Y., Schnall-Levin, M., Wyatt, P. W., Hindson, C. M., Bharadwaj, R., Wong, A., Ness, K. D., Beppu, L. W., Deeg, H. J., McFarland, C., Loeb, K. R., Valente, W. J., Ericson, N. G., Stevens, E. A., Radich, J. P., Mikkelsen, T. S., Hindson, B. J., and Bielas, J. H. (2017). Massively parallel digital transcriptional profiling of single cells. *Nature Communications*, **8**(1), 14049.
